# Impact of the COVID-19 pandemic on an interdisciplinary endoscopy unit in a German “hotspot” area: a single center experience

**DOI:** 10.1007/s00464-020-08119-w

**Published:** 2020-11-02

**Authors:** Dörte Wichmann, Naushad Bijoy Atique, Dietmar Stüker, Stefano Fusco, Ulrike Schempf, Julia M. Grottenthaler, Michael Böckeler, Christian Thiel, Lars Zender, Alfred Königsrainer, Nisar P. Malek, Christoph R. Werner

**Affiliations:** 1 Department of General, Visceral and Transplantation Surgery, Interdisciplinary Endoscopy Unit, University Hospital Tübingen, Hoppe-Seyler-Strasse 3, 72076 Tübingen, Germany; 2 Department of Internal Medicine VIII, Pneumology, University Hospital Tübingen, Ottfried-Müller-Strasse 14, 72076 Tübingen, Germany; 3 Department of Gastroenterology, Hepatology, Gastrointestinal Oncology, Geriatrics and Infectious Diseases, University Hospital Tübingen, Otfried-Müller-Strasse 10, 72076 Tübingen, Germany; 4 Central Operation Theatres at the University Hospital Tübingen, Hoppe-Seyler-Strasse 3, 72076 Tübingen, Germany

**Keywords:** COVID-19, Interdisciplinary endoscopy, Emergency bleeding situation, Prevention of infection

## Abstract

**Background and study aims:**

Since December 2019, the severe acute respiratory syndrome coronavirus 2 (SARS-CoV-2), the causative pathogen of coronavirus disease 2019 (COVID-19), has posed a pandemic threat to global health and has challenged health care system in all affected countries.

**Patients and methods:**

This is a combined study including a descriptive part about the changes in the daily work routine of an Interdisciplinary Endoscopic Unit (IEU) and a prospective analysis of patients tested positive for SARS-CoV-2 who required endoscopic interventions. Conclusively, we present the finding of a point-prevalence analysis in the staff of the IEU.

**Results:**

We present effects of the COVID-19-related restructuring of processes in our interdisciplinary endoscopy unit (IEU) with respect to cancelation of examinations, relocation of staff to other departments, impact of SARS-CoV-2 on medical staff of the IEU, and supply of protective clothing. Additionally, we analyzed the cohort of COVID-19 patients: Sixteen endoscopic interventions were done in ten patients. In all patients with confirmed infection with SARS-CoV-2, emergency endoscopies were required for relevant bleeding situations. Re-endoscopies were required only in critically ill COVID-19 patients.

**Conclusions:**

The restructuring of processes in the IEU was feasible in short time, effective, and can also be applied broadly at least in developed countries [Garbe et al. in Gastroenterology 159:778–780, 2020; Repici A, Pace F, Gabbiadini R, Colombo M, Hassan C, Dinelli M, Group IG-CW, Maselli R, Spadaccini M, Mutignani M, Gabbrielli A, Signorelli C, Spada C, Leoni P, Fabbri C, Segato S, Gaffuri N, Mangiavillano B, Radaelli F, Salerno R, Bargiggia S, Maroni L, Benedetti A, Occhipinti P, De Grazia F, Ferraris L, Cengia G, Greco S, Alvisi C, Scarcelli A, De Luca L, Cereatti F, Testoni PA, Mingotto R, Aragona G, Manes G, Beretta P, Amvrosiadis G, Cennamo V, Lella F, Missale G, Lagoussis P, Triossi O, Giovanardi M, De Roberto G, Cantu P, Buscarini E, Anderloni A, Carrara S, Fugazza A, Galtieri PA, Pellegatta G, Antonelli G, Rosch T, Sharma P (2020) Endoscopy units and the COVID-19 Outbreak: a Multi-Center Experience from Italy. Gastroenterology;]. The endoscopy-related rate of SARS-CoV-2 infection of staff is low, but supply of protective equipment is crucial for this. Endoscopic procedures in COVID-19 patients were not directly related to SARS-CoV-2 infection, but to other underlying diseases or typical complications of long-term ICU treatment.

The current SARS-CoV-2 pandemic has a direct impact on the social life and on health systems of all affected countries. Rapid and fundamental changes have become necessary in order to respond adequately to the pandemic. The countries first affected by SARS-CoV-2, China, South Korea, and Italy, have implemented far-reaching social restrictions (“lockdown”). However, in some of these countries, the pandemic drastically tested and in some cases overstretched the health care systems [3]. Thus, the countries that were affected after these first countries were warned and had a longer lead time to respond to the pandemic.

In Germany, the first proven infection with the novel coronavirus SARS-CoV-2 was detected on 27th January 2020 as an “imported” infection from China [4]. A first, localized outbreak occurred in the Heinsberg district in connection with a carnival event [case cluster]. More widespread increases in newly diagnosed SARS-CoV-2 infections followed from the beginning of March 2020, particularly in southwest Germany and Bavaria, presumably after many SARS-CoV-2 positive returnees from their winter holidays who became infected in the Alpine region at the end of February were diagnosed [5].

The first two cases of SARS-CoV-2 infection detected in the district of Tübingen were diagnosed on 26th February 2020; the infection was “imported” from Milan. In Tübingen, health care providers were immediately affected, since “patient No. 1” was a physician of the staff of the University Hospital Tübingen. The district of Tübingen was one of the “hotspots” in Germany during the peak phase of the first Corona wave at the end of March and beginning of April 2020 with an infection rate of 422/100,000 inhabitants (8th April, 9th place nationwide; see Fig. 1; for comparison: Germany 124/100,000 inhabitants (www.rki.de, 08.04.2020).

**Fig. 1:**
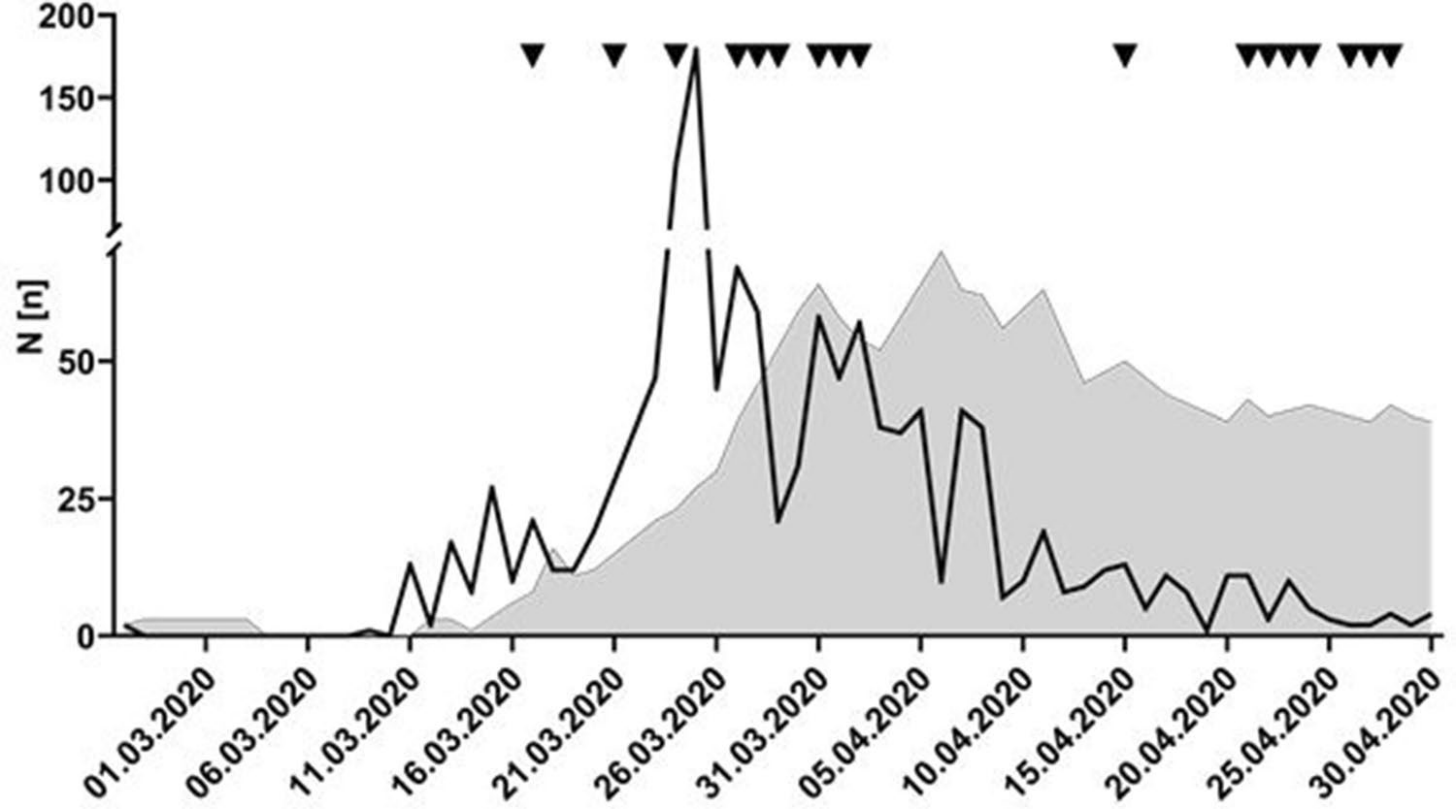
Timeline of the development of the 2020 SARS-CoV-2 pandemic in the district of Tübingen, Baden-Württemberg, Germany, and number of patients with conducted SARS-CoV-2 infections at the University Hospital of Tübingen

In Germany, extensive measures have been taken to break chains of infection. Already on 13th March 2020, the decision of the Federal Government and the federal states to indefinitely postpone elective medical examinations and operations heralded the start of restructuring measures in the German healthcare system. On 16th March 2020, school and kindergartens were closed. In particular, a “Kontaktverbot” (the German variant of a “lockdown”) was decreed by all federal states between 20th March and 22nd March 2020, which included the closure of many shops, some large factories, and a restriction in personal contacts.

As a response of the specialist societies to the COVID-19 pandemic, on 18th March 2020, the European Society of Gastrointestinal Endoscopy (ESGE) issued recommendations for the handling of COVID-19 patients in endoscopy units, which were adopted by the German specialist society DGVS (German Society for Gastroenterology, Digestive and Metabolic Diseases) [6]. Also on 18th March 2020, the German Society for Pneumology and Respiratory Medicine (DGP) published recommendations for the use of bronchoscopy in times of the COVID-19 pandemic [7, 8].

This article is intended to provide an overview of the impact of the COVID-19 pandemic on an interdisciplinary endoscopy unit (IEU, gastroenterological endoscopy, surgical endoscopy, and bronchoscopy) at the University Hospital Tübingen, a center for tertiary care in South-western Germany. First, preparations for COVID-19 pandemic with respect to the IEU are presented, and compared with those of other endoscopy units [1, 2]. Additionally, the direct impact of SARS-CoV-2 on the staff of the IEU will be shown, together with a point-prevalence analysis of SARS-CoV-2 of the staff at the end of the SARS-CoV-2 wave in Tübingen. Finally, to our knowledge for the first time, we conducted a prospective single center analysis of the patients infected with SARS-CoV-2, which required a diagnostic or therapeutic endoscopic intervention.

## Materials and methods

### Study design

This is a combined study including a descriptive part about the preparations for COVID-19 and the impact of COVID-19 on the daily routine of the work in an Interdisciplinary Endoscopic Unit (IEU), and a point-prevalence analysis for possible positive SARS-CoV-2 carriers among the staff of the IEU, and finally a prospective analysis of SARS-CoV-2 positive patients who required endoscopic intervention.

Results are presented in the captures: (a) SARS-CoV-2-pandemic-related restructuring of the processes in the IEU, (b) SARS-CoV-2-pandemic-related impact on the staff of the IEU, and (c) analysis of endoscopic interventions in patients with confirmed or suspected SARS-CoV-2 infection.

The local ethics committee of the University Hospital of Tübingen, Germany, approved this study (AZ: 242/2020BO2) and by ClinicalTrials.gov (NCT04423003). Investigation period was March and April 2020. Informed consent for endoscopy was obtained from all individual participants or from their juridical persons.

The point-prevalence analysis (PPA) was conducted in all employees of the IEU at the end of the investigation period. Testing was done by the corporate medical service of the University hospital of Tübingen.

### Database

Data are available in SPSS v. 24.0.0.1 (IBM, Armonk, NY, USA) and were presented as mean ± SD.

## Results

### SARS-CoV-2-pandemic-related restructuring of the processes in the IEU

#### Cancelation of interventions

**Fig. 2:**
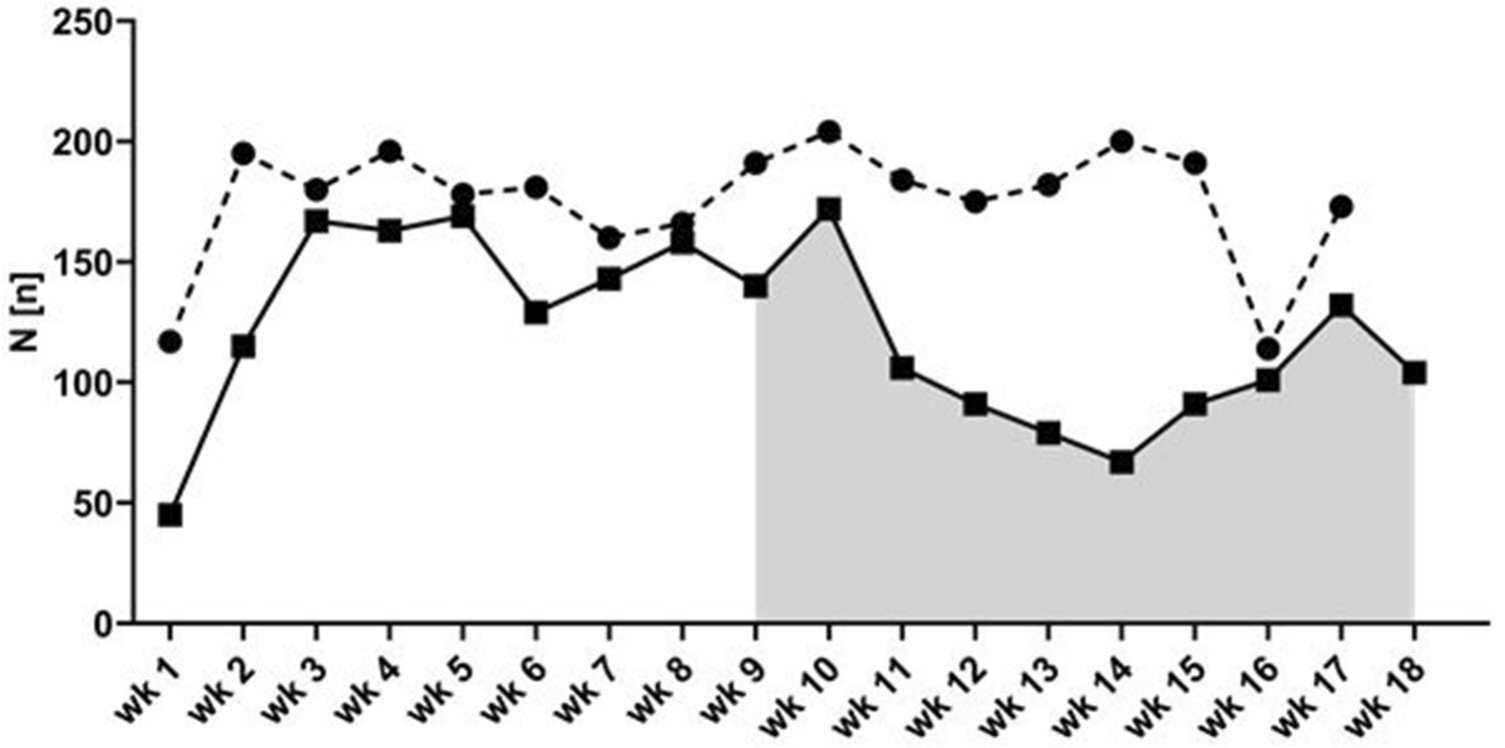
Endoscopic examinations per week in 2019 (dashed line) and 2020 (black line). The gray area indicates the period of the SARS-CoV-2 pandemic in the district of Tübingen

From 16th March 2020 onwards, elective examinations were removed from our examination program or postponed to a preliminary later date (end of April and beginning of May). The decision as to which formally elective procedures could still be performed despite the SARS-CoV-2 lockdown was made by an expert board. They included preventive measures such as ligature therapy of esophageal varices after variceal bleeding, or diagnosis of suspected cancer. This resulted in a significant decrease in the number of patients in the IEU. The number of examinations fell in calendar weeks 11–18 (96/ week) to 68% of the weekly average in comparison to calendar weeks 1–10 (140/ week, with a nadir of 67/ week) (see Fig. 2).

#### Separation of patients at risk and procedural measures

From 17th March 2020, a separate room in the endoscopy unit was dedicated for patients with COVID-19 infection where they would be examined under anesthesia (with or without intubation anesthesia), with the provision that only examinations with the most urgent priority (emergency procedures) would be performed on these patients. Intensive care unit patients with COVID-19 should preferably be examined in the intensive care unit wing reserved especially for COVID-19 patients (last max. 40 beds). For this purpose, a special endoscopy tower was equipped, which remained in the intensive care unit dedicated for COVID-19 patients to keep the risk of contamination in endoscopy as low as possible. Being the endoscopic examination with the highest risk of aerosol formation, for bronchoscopy, in all patients the use of FFP-2/N95 masks or equally protective equipment became compulsory. Patients for bronchoscopy were transferred directly to and from the bronchoscopy room to the respective wards to avoid dissemination of aerosols in our holding room and wake-up area by coughing patients.

After some time in advance, from 27th March 2020, a negative pressure room could be kept in a separate operating wing, in which endoscopic examinations of COVID-19 patients, who were not in intensive care, could be carried out. It was then interdisciplinary consented to conduct endoscopic examinations in COVID-19 patients under general anesthesia to minimize formation of aerosols. Examinations with fluoroscopy (ERCP, PTCD) should continue to be carried out in the fluoroscopy room of the endoscopy unit. During the observation period, one endoscopic examination in a confirmed SARS-CoV-2 positive patient (Pat.-No. 2) was realized in the negative pressure room. All other endoscopies were done at the intensive care unit.

It was consented informally to reserve endoscopic examinations in SARS-CoV-2 infected patients for nurses and physicians of younger age (< 50 years) or voluntaries, since it was known, that COVID-19 had a poorer outcome in older infected persons.

### SARS-CoV-2-pandemic-related impact on the staff of the IEU

#### Redistribution of medical staff from the IEU to COVID-19 wards

Along with the reduction of elective procedures in the IEU, the hospital was prepared to admit COVID-19 patients, which meant the infectious diseases ward was gradually expanded from 10 to 40 beds and the gastroenterological ward was reduced from 30 to zero beds. For compensation, the geriatrics ward of the department became a mixed ward for all non-COVID-19 patients, and the other departments of the Internal medicine and the surgery department were opened to patients with gastroenterological diseases. Additionally, a separate wing of the ICU was dedicated for admission of COVID-19 patients (40 beds).

The nursing staff and physicians of the IEU freed up by the capacity restrictions were to be distributed among COVID-19 wards. Thus, 2 out of 11 (18%) physicians and 3 out of 18 (17%) nurses and other staff were withdrawn from the IEU relatively to the beginning of SARS-CoV-2 activity. This meant a reduction of physician staff to 72% and of nurses and other medical staff to 83%.

Since the number of hospitalizations during the SARS-CoV-2 pandemic in Tübingen did not exceed the capacities of the new COVID-19 wards, no further redistribution of staff at least from the IEU was necessary. However, a phased plan was already developed on 18th March 2020, which would ultimately have included a core team of 3 physicians and 7 nurses (out of 11 physicians and 18 nurses and other staff) to run the Endoscopy unit; so that two teams could have operated independently during the daytime service and emergency services could have been provided at night.

#### SARS-CoV-2 infections, quarantine, and SARS-CoV-2 point-prevalence study in the staff of the IEU

Two out of 11 (18%) physicians of the IEU were tested positive for SARS-CoV-2, one of them had a very mild course of disease and resumed his work after 14 days, and the other had more severe symptoms and was not symptom free for more than 14 days. Both physicians worked partly in the IEU and partly on the Department of Pneumology, but had no contact in the IEU with patients with proven SARS-CoV-2 infections.

Due to quarantine regulations, 1 out of 11 (9%) physicians of the IEU had to undergo 14-day quarantine, being an unprotected contact person for an infected medical colleague, who was “patient No. 1”. The same physician was later freed from duty for the duration of the SARS-CoV-2 pandemic as a member of a special risk group.

At the end of the observation period (April 22th and 23th), a point-prevalence analysis was performed among the medical staff of the IEU. The test was done via pathogen detection of SARS-CoV-2 nucleic acid from throat swabs. In total, 8 out of 11 physicians and 14 out of 18 members of the endoscopic assisting staff were tested. None of the persons tested were positive for SARS-CoV-2. Both physicians from the Department of Pneumology, who were tested positive for SARS-CoV-2 at the end of March, were also tested negative in the point-prevalence analysis at the end of April.

#### Supply of protective clothing

A crucial problem during the COVID-19 pandemic was the lack of protective clothing, especially for medical personnel, but also for the general population. Ultimately, this shortage has mainly manifested itself in a lack of FFP-2 and -3/N95 masks, splash shields and protective goggles. The situation was remedied by rationing and extending the wearing times of FFP-2 and -3/N95 masks; additionally, splash shields were rare. This ultimately caused a reduction in safety standards. The undersupply of protective equipment at the peak of the SARS-CoV-2 wave in Tübingen (and presumably also in Germany) did not become more critical, probably only because there was not such a mass of hospitalizations as in other countries such as China, Italy, Spain, USA, and France at their respective peaks of the waves. Protective gowns and aprons were not in short supply and were not rationed in Tübingen.

Bronchoscopy in particular is a high-risk intervention in terms of aerosol formation and should have been performed with FFP-2/N95 masks as standard even for patients without known SARS-CoV-2 infection, at least during the COVID-19 period [7, 8]. However, there was no capacity for this, certainly not if the mask had to be changed after each patient.

**Fig. 3:**
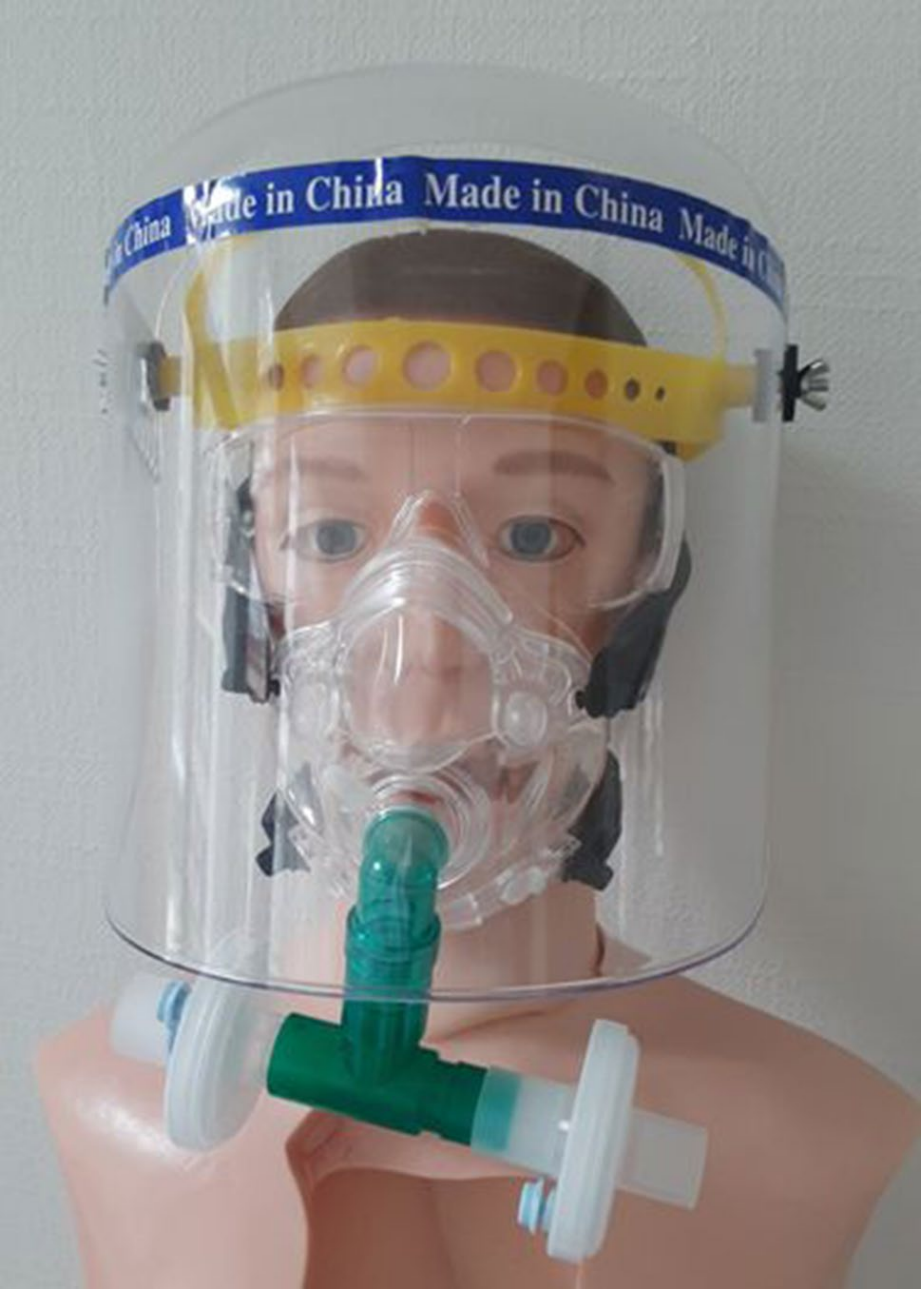
Personal protective clothing used during the high time of the SARS-CoV-2 Pandemic in Tübingen: CPAP masks (Hans Rudolph Inc., Shawnee, KS, USA), a T-piece (Cirrus nebulizer breathing system® T-kit 22 mm, Intersurgical Ltd., Wokingham, Berkshire, UK), and 2 bacterial/ viral filters (medisize®, Meditera, Tire, Izmir, Turkey)

To remedy this situation, by initiative of our colleagues of the Department of Pneumology, a reusable respiratory mask was developed from CPAP masks (Hans Rudolph Inc., Shawnee, KS, USA), a T-piece (Cirrus nebulizer breathing system® T-kit 22 mm, Intersurgical Ltd., Wokingham, Berkshire, UK) and 2 bacterial/ viral filters (medisize®, Meditera, Tire, Izmir, Turkey), which could also be used by people wearing glasses (Fig. 3). The mask could be disinfected, the filters were changed every working day or always after use in a SARS-CoV-2 positive patient. The mask was approved by the Department of Hospital Hygienic. During all bronchoscopy and endoscopic GI examinations involving SARS-CoV2-positive patients the whole staff present in the room used the CPAP masks. For bronchoscopies we did not difffer between SARS-CoV2-positive or negative patients, every bronchoscopy was done with the masks.

These masks were used as standard in bronchoscopy from 6th April 2020 on, in GI endoscopy only in the case of a SARS-CoV-2 positive patient. As a supplement to simple protective glasses, a set of splash protection visors was purchased via the worldwide-web, which was used from 12th April 2020 on. However, since purchase was not in line with the procurement regulations of our hospital, the refunding of the costs was denied by the administration. Importantly, the splash masks, which were ordered in parallel in-house in accordance to procurement regulations, were not delivered until now (May/June 2020).

### Analysis of endoscopic interventions in patients with confirmed or suspected SARS-CoV-2 infection

We analyzed the endoscopic interventions between March 1th and May 5th 2020 in patients with confirmed or suspected SARS-CoV-2 infection. Overall, sixteen endoscopic interventions were done in nine SARS-CoV-2 positive tested patients (m:f = 5:4, mean age 62.44 years ± 15.44), and one endoscopic examination was indicated in a patient with suspected SARS-CoV-2 infection (f, 65 years old). Patient characteristics are documented in Table 1. In five patients, COVID-19 disease with a critical acute respiratory distress syndrome was proven (Pat.-No.1, 4, 7, 8 and 9). Patient No. 4 was confirmed positive for SARS-CoV-2 before the hospital admission; repeated tests during this time as inpatient were negative. In patient No. 6, SARS-CoV-2 infection resulted in a relevant kidney failure and repeated duodenal ulcerations with bleeding episodes. In patients No. 2 and 5 both tested positive for SARS-CoV-2, no respiratory or other infection-related symptoms were presented.

**Table 1:** Patient characteristics, the bold marked patients died during the examination period

Pat.-No	Sex	Age	Positive tested for SARS-CoV-2	Symptoms for COVID-19	Causative Indication for endoscopy	APACHE II at examination date	SOFA Score	Invasive ventilation before endoscopy
**1**	**♂**	**56**	**Yes**	**Yes**	**Bleeding**	**31**	**19**	**Yes**
2	♂	60	Yes	No	Bleeding	No ICU	7	No
**3**	**♀**	**63**	**Yes**	**Yes**	**Bleeding**	**15**	**7**	**No**
**4**	**♂**	**59**	**Yes**	**Yes**	**Bleeding**	**28**	**17**	**Yes**
**5**	**♀**	**83**	**Yes**	**No**	**Bleeding**	**No ICU**	**10**	**No**
6	♂	80	Yes	No	Bleeding	18	15	No
7	♂	50	Yes	Yes	Bleeding	11	15	Yes
8	♀	79	Yes	Yes	Bleeding	28	27	Yes
9	♂	32	Yes	Yes	Bleeding	23	19	Yes
10	♀	65	No	No	Cholestasis	No ICU	8	No

In addition, in one patient with known contact to a SARS-CoV-2 positive person who required endoscopic interventions, all protective measurements were used as in a case of confirmed infection (Pat.-No.10).

In all patients with confirmed infections with SARS-CoV-2, emergency endoscopies were required for relevant bleeding situations. In five patients, bleeding sources were located in the upper gastrointestinal tract. In one patient, the bleeding source was located in the lower gastrointestinal tract, and in two patients, relevant bronchial bleedings were detected. Severely ill COVID-19 patients required supportive therapy with lung and kidney replacement, all patients were treated with anticoagulative therapies.

We listed blood tests including coagulation status at the time of endoscopic interventions in Table 2.

**Table 2:** Laboratory parameters at the date of the initial endoscopic examination, the bold marked patients’ died during the examination period

Pat.-No	Hemoglobin (g/dl)	Hematocrit	WBC (n/ml)	Platelets (n × 10^3^/ml)	INR	CRP (mg/dl)
**1**	**6.4**	**23**	**2930**	**74**	**1**	**21.1**
2	7.1	19.8	8870	96	1.1	13.69
**3**	**9.5**	**31**	**21,390**	**408**	**1**	**5.98**
**4**	**6.5**	**19.2**	**13,670**	**82**	**1.4**	**14.57**
**5**	**7.7**	**24.9**	**5140**	**169**	**1.2**	**6.38**
6	7.2	20.4	9620	92	1.6	26.26
7	6.8	19.6	3170	39	1.2	10.47
8	7.2	21.6	10,770	77	1	6.91
9	6.5	20.3	15,420	209	1.3	18.68
10	9.6	28.3	6990	251	1	12.75
Mean	7.5	22.81	9797	149.7	1.18	13.67

In five patients, re-endoscopies were required. All of these patients were critically ill with confirmed infections of COVID-19 Endoscopic examinations were realized at ICU (*n* = 14) and in an OR with negative pressure (*n* = 1). At time of endoscopic interventions, all patients were under general anesthesia and intubated. Patient No. 10 was examined in the rooms of the IEU for an ERC. Characteristics of the endoscopic interventions are listed in Table 3.

**Table 3:** Characteristics of endoscopic interventions in all confirmed SARS-CoV-2-positive patients’ (i/v = intubated and ventilated)

Pat.-No	Procedure	Intervention	Technical success of the intervention	Redo endoscopies	i/v prior the endoscopy
**1**	**Gastroscopy**	**2 Hemoclips, injection of fibrin glue**	**Yes**	**2**	**Yes**
2	Gastroscopy	No	No intervention	1	No
**3**	**Gastroscopy**	**No**	**No intervention**	**1**	**Yes**
**4**	**Gastroscopy, Colonoscopy**	**No**	**No intervention**	**0**	**Yes**
**5**	**Gastroscopy, Colonoscopy**	**2 Hemoclips, injection of fibrin glue**	**Yes**	**0**	**No**
6	Gastroscopy	2 Hemoclips, injection of fibrin glue	Yes	2	No
7	Gastroscopy	2 Hemoclips	Yes	1	Yes
8	Bronchoscopy	Lavage, Cryobiopsy	Yes	0	Yes
9	Bronchoscopy	Lavage, Cryobiopsy	Yes	0	Yes

Bold marked patients’ died during the examination period

Overall, four seriously ill COVID-19 patients died in the period of this study (44.44%, Pat.-No.: 1, 3, 4, 5; m:f = 2:2, mean age 65.25 years ± 9.43). None of these patients died of hemorrhage but on multiple organ failure caused by COVID-19.

Patient No. 2 was discharged after a hospital stay in total of 14 days. Patient No. 6 was successfully transferred from ICU to a standard care unit. The remaining patients (Pat.-No.: 7, 8 and 9) are still treated at our ICU.

Patient No. 10, who was tested negative for SARS-CoV-2 after contact to a positive tested person, was discharged regularly and is in continuous treatment as an outpatient.

In relation to the course of the pandemic in the district of Tübingen, two peaks of incidence of endoscopical examinations could be noticed (Fig. 1): the first peak in parallel with the peak of newly infected patients, thus probably representing the high incidence of SARS-CoV-2 infection in the population, and a second peak at the end of the pandemic in the district of Tübingen.

## Discussion

At the end of the first wave of the COVID-19 pandemic in Germany—and in one of the “hotspot” areas, Tübingen, we can resume that Germany experienced a milder course of the pandemic with respect to the number of deaths, while in other regions of the world the disease still takes its toll [5].

The response of the German health care system indisputably was quick and fundamental, by restructuring capacities and focusing on the COVID-19 threat. In this prospective analysis, we concentrated on the response in an interdisciplinary endoscopy unit of a center for tertiary care, the University Hospital Tübingen.

To expand capacities for COVID-19 patients, the nursing and physician staff of the IEU was reduced down to 83%, and 73%, respectively. As in the IEU in Tübingen, in most endoscopy units in Germany relocation of staff was expected [1]. In Northern Italy, 65.9% of centers relocated physicians and 75.6% of centers relocated nurses to other departments [2].

For the IEU in Tübingen, this meant reductions in elective endoscopic examinations to 68%, with a nadir of 67 endoscopic examinations/ week at the high time of SARS-CoV-2 activity in the district of Tübingen. In multi-center surveys in Germany and Northern Italy, all or almost all endoscopy units reduced their routine program to a certain level, in Germany most endoscopy units reduced their program to 40%-60% [1]. In Northern Italy, a region which was severely affected by the pandemic, most centers reduced their program to 1–25% [2].

To avoid intraprocedural transmission of SARS-CoV-2, the IEU in Tübingen could use a negative pressure room for examination of COVID-19 patients. In a multi-center survey, just 20.3% of centers were able to provide complete separation for COVID-19 patients in Germany [1]. Accordingly, in Northern Italy 7 out of 42 (17.1%) centers could provide a negative pressure room as recommended by the ESGE [2, 6]

SARS-CoV-2 took a toll on the staff of the IEU in Tübingen: 9% (1/11) of physicians were freed from duty due to quarantine or for protective measures. 18% (2/11) of staff became infected with SARS-CoV-2 and developed COVID-19, but the source of infection most likely was outside of the IEU. In other regions medical staff also was affected: in China, more than 3,300 medical staff were infected (4% of the 81,285 reported infections). In Spain, nearly 6,500 medical personnel were infected (13.6% of the country’s 47,600 total cases, 1% of medical personnel; 25th March 2020) [9]. In Northern Italy, 12 out of 42 endoscopy units had confirmed SARS-CoV-2 infections in endoscopy staff, with 6 persons requiring hospitalizations [2]. As in our center, no transmission directly related to endoscopic procedures in COVID-19 patients was reported there [2, 10]. At the end of the observation period, a point-prevalence study of the IEU staff was conducted, with no detection of SARS-CoV-2.

Though the restructuring of processes in the hospital and in the IEU were effective, at the high time of the pandemic a crucial part of effective care of COVID-19 patients became rare: protective clothing. Therefore, the successful restructuring of the German health care system would have been jeopardized by lack of a “penny item”, which could have possibly led to a collapse due to SARS-CoV-2 infected medical staff. In the German survey, most heads of the endoscopy units expected shortage of personal protective equipment as likely or very likely [1]. In the future, in the eve of a possible second wave of SARS-CoV-2 or a permanent “low-level” prevalence of COVID-19, one focus of the authorities should be the storage and local production of crucial protective gear, even if it may seem not to be cost-effective at first sight.

Since the infrastructure for the examination of SARS-CoV-2 positive patients has been set up already, we decided to keep it on duty to face the possible second wave of SARS-CoV-2 until the risk of infection is minimized by an effective vaccination or other measures. Furthermore the establishment of the negative pressure endoscopy suite is also suitable for future patients with other infectious airway diseases.

With respect to SARS-CoV-2 infected patients with endoscopic examinations, GI bleeding was the main indication for endoscopy in our center, thus reflecting our restrictive policy to only conduct emergency endoscopies in these patients [11]. In our center 16 endoscopic interventions were done in 9 COVID-19 patients. In Northern Italy, in 41 centers, altogether 35 endoscopic procedures were performed in COVID-19 patients [2]. In our center, two phases of higher frequency of endoscopic examinations could be observed: First, in the high time of new infections, patients with a need for endoscopic treatment in first line, and a concomitant infection or suspected infection with SARS-CoV-2 in second line underwent endoscopy. Later on, mainly patients with typical complications of long-term critical illness were in need of endoscopic therapy (see Table 2, and Fig. 1). Especially patients treated with extracorporeal membrane oxygenation (ECMO) machines and/or continuous renal replacement therapy (CRRT) with anticoagulatory treatment developed bleeding situations [3, 12]. This finding could reflect the shift in demographics of the pandemic with a peak phase of broadly spread infections in the beginning and, later on, in the time of fewer new infections, a smaller group of long-term critically ill patients at our ICU.

In conclusion, the restructuring of processes in our IEU was feasible in short time, effective and can also be applied broadly at least in developed countries [1, 2]. The endoscopy-related rate of SARS-CoV-2 infections of staff is low [2, 10](in our IEU zero), but supply of protective equipment is crucial for this. Endoscopic procedures in COVID-19 patients were not related to SARS-CoV-2 infection, but to other underlying diseases or typical complications of long-term ICU treatment.
